# Ophthalmology and Otology

**Published:** 1893-05

**Authors:** Alvin A. Hubbell

**Affiliations:** Buffalo, N. Y.


### OPHTHALMOLOGY AND OTOLOGY.

By ALVIN A. HUBBELL, M. D., Buffalo, N. Y.

### TREATMENT OF MARGINAL BLEPHARITIS WITH SOLUTIONS OF CORROSIVE SUBLIMATE IN GLYCERINE.

Dr. L. Borno (*The Medical Week*, January 13, 1893,) recommends the application of a solution of corrosive sublimate in glycerine as the best treatment for blepharitis. It is the method employed by Dr. Despaquet, of Paris, in the treatment of this troublesome disease, which is not easily controlled by the ordinary means at our disposal. It consists in painting the outside of the margins of the lids, close to the roots of the eyelashes, with 1-30 and 1-100 solutions of perchloride of mercury in glycerine.

The stronger solution is applied every second day *by the surgeon*, after the eyelashes and scabs have been removed. At the end of each application the part is dried with a piece of cotton-wool. The pain produced by the passage of some of the lotion into the eye is relieved by bathing with cold water, and is much less acute than one would expect from the strength of the solution.

This is due to the fact that, like carbolic acid, corrosive sublimate loses some of its causticity in glycerine.

The 1-100 solution is painted on daily by *the patient himself.*

According to Dr. Borno, the condition of the lids shows a marked improvement from the very first week of the treatment. A cure is obtained within two months even in inveterate cases of blepharitis, with thickening of the margins of the eyelids.

---

### TREATMENT OF TRACHOMA BY EXPRESSION.

Dr. H. Knapp (*Archives of Ophthalmology*, January, 1893,) contributes another communication on the treatment of trachoma by expression with his roller forceps. He sums up as follows:

1. Rapid, perfect, and permanent recovery by expression alone, or expression followed by a short course of mild caustic treatment, takes place in the majority of cases. This refers chiefly to the cases of pure follicular trachoma.

2. Imperfect recovery, *i. e.*, disappearance of trachoma, but having more or less shrinkage of the conjunctiva, is the common issue of old, neglected cases of inflammatory trachoma.

3. Relapses occur in both simple and inflammatory trachoma. They have been recorded in ten cases of the first series, and in eight of the second. The majority of patients came back with a relapse after having felt comfortable and done nothing for their eyes many months after the operation. The relapses were mostly promptly cured by a second expression; in a few cases a third was necessary. In two cases the steady after-treatment with the copper crystal did not prevent a relapse. They had been treated as out-patients and worked in a dusty atmosphere.

4. The operation has neither destroyed nor injured an eye.

---

### SYMPTOMS OF CEREBRAL IMPLICATION IN OTORRHEA.

G. P. Field, in his lecture before the Harveian Society of London, (*British Medical Journal*, December 17, 1892,) says that the pathology of brain disease, due to otorrhea, is usually quite simple. An outbreak of head-symptoms is usually only the final stage in a long series of insidious morbid changes, of which the patient himself is unaware. The mere accumulation of pus within the tympanum may give rise to symptoms simulating those of basal meningitis. The special sign of actively inflammatory brain lesion from aural suppuration, is pain. Almost universally there are dull achings over and radiating from the mastoid region, and racking headache. Oscillations between subnormal and high temperature with rigors indicate pyemia. The temperature in uncomplicated meningitis is continuously high, as also in simple aural abscess, in which, however, there are modifications, according to complications present. An abrupt fall in temperature after an initial rise signalizes the completion of the development of an abscess; the temperature then, as a rule, remains at or below the normal.

Other diagnostic signs of cerebral abscess are slow, full, and even pulse, constipation (as in meningitis), sluggish cerebration, respirations which are rythmical, but less in number and extent, and localized paralysis and convulsive movements. In meningitis there is usually delirium, and the pulse is quick, small, and irregular.

Even when the diagnosis is almost certainly that of diffuse meningitis, the perforation of the mastoid cells and tympanic wall and exploratory trephining by Wheeler's operation may be the means of saving life.

---

### EXPLORATORY OPERATIONS FOR INTRACRANIAL ABSCESS FROM AURAL DISEASE.

THE Year-Book of Treatment, for 1893, records that Wheeler was the first surgeon to call attention to the desirableness of making one hole in the skull instead of two or three, as practised by Barker and others. Wheeler's plan is, however, to do this by an extension of the necessary mastoid operation, by removing the bone upwards and forwards to get at the middle fossa of the cranial cavity, and backwards and downwards to expose the lateral sinus and posterior fossa. In this form of operation it will be observed that the temporo-sphenoidal lobe and lateral ventricles are explored from a spot which is much lower than that recommended by Barker and Birmingham, for the former recommends that the cerebrum should be examined from a quarter-inch trephine hole, having its center one and a quarter inches behind and above the center of the external auditory meatus.

Truckenbrod (*Monatsh. für Ohrenheilkunde,*) has described a case in which unequivocal signs of cerebral abscess followed otitis media. The operation consisted in chiselling open the left mastoid antrum, and removal of the tegmen antri, which separates the antrum from the cranial cavity, incision of the membranes which were found adherent to the brain, and exploring the temporo-sphenoidal lobe with a syringe inserted inwards, forwards, and upwards. Pus was found and the opening was enlarged by incision, and drained. The case recovered.

Dean (*Lancet*, July 30, 1892,) operated on a case suffering from headache, drowsiness, and optic neuritis, following chronic suppuration of the middle ear, together with mastoid inflammation. He opened the skull with a three-quarter inch trephine, setting the pin one and a quarter inches behind and a half inch above the center of the auditory meatus. This exposed the lateral sinus below and the dura mater over the temporo-sphenoidal lobe above. These were explored without finding pus. The opening in the skull was then enlarged downwards and backwards by cutting forceps, exposing the dura mater of the posterior fossa. The dura was incised and the cerebellum explored with a trocar, when pus was found and flowed freely. A rubber drainage-tube was inserted and the patient made a good recovery. Dean urges the importance of exploring the different areas likely to be affected when once the skull is opened.

Birmingham (*Dublin Journal of Medical Science*, February, 1891,) has suggested certain guides for trephining for certain areas:

Lateral sinus, one and one-quarter inches behind the center of the bony meatus, and on a level with its upper border.

Temporo-sphenoidal lobe, one and one-quarter inches behind and two inches above the center of the bony meatus.

Mastoid antrum, one-twelfth of an inch above the level and behind the center of the bony meatus ; depth, three-fifths to three-fourths of an inch.

Cerebellum, two inches behind the center of the bony meatus and one inch below Reid's base line. [An imaginary line drawn from the occiput through the center of the external auditory meatus to the lower angle of the orbit.—A. A. H.]

---

AT THE Clinical Society of London, Dr. de Haviland Hall reported a case of rhinolith. The stony mass occurred in the left nostril of a girl of sixteen, who had been troubled with a fetid discharge since the age of two and one-half years. The stone had been removed piecemeal by means of forceps, and was found to consist of organic matter, 26.4, and calcium phosphate, 73.6, and weighed 92 grains. Rhinoliths are comparatively rare, and the best account of them is to be found in a monograph by Dr. Max Seeligmann, of Carlsruhe.—*Denver Medical Times.*

